# Draft Genome Sequence of *Nonomuraea* sp. TP-A0861, a Producer of Myxochelin A

**DOI:** 10.1128/genomeA.01430-15

**Published:** 2015-12-10

**Authors:** Hisayuki Komaki, Natsuko Ichikawa, Akira Hosoyama, Nobuyuki Fujita, Yasuhiro Igarashi

**Affiliations:** a Biological Resource Center, National Institute of Technology and Evaluation (NBRC), Chiba, Japan; b NBRC, Tokyo, Japan; c Biotechnology Research Center and Department of Biotechnology, Toyama Prefectural University, Toyama, Japan

## Abstract

*Nonomuraea* sp. TP-A0861 produces the nonribosomal peptide myxochelin A, which is known as a microbial siderophore. Here, we report its draft genome sequence. The genome contains at least three nonribosomal peptide synthetase gene clusters, one of which is proposed to be responsible for the biosynthesis of myxochelin A.

## GENOME ANNOUNCEMENT

Myxochelin A is a microbial siderophore, originally isolated from a myxobacterium (1). Its biosynthetic pathway was reported in 2008 (2). In our screening program on secondary metabolite diversity of endophytic actinomycetes, *Nonomuraea* sp. TP-A0861 isolated from a soybean root was found to produce myxochelin A (3). This was the first discovery of myxochelin A from actinomycetes. In the present study, the whole-genome shotgun sequencing of the strain was performed, and nonribosomal peptide synthetase (NRPS) and polyketide synthase (PKS) gene clusters were surveyed to evaluate the biosynthetic potential of strain TP-A0861. A plausible biosynthetic gene cluster for myxochelin A is proposed.

*Nonomuraea* sp. TP-A0861 was deposited to the NBRC culture collection (NBRC 110462). The whole genome of the *Nonomuraea* sp. TP-A0861 monoisolate was read by using a combined strategy of shotgun sequencing with GS FLX+ (Roche) (83-Mb sequences, 9-fold coverage) and paired-end sequencing with MiSeq (Illumina) (881 Mb, 98-fold coverage). These reads were assembled using Newbler version 2.8 software, and subsequently finished using GenoFinisher software (4), which led to a final assembly of eight scaffold sequences of >500 bp each. The total size of the assembly was 8,992,127 bp, with a G+C content of 72.5%. Coding sequences were predicted by Prodigal (5), and PKS and NRPS gene clusters were searched for as previously reported (6).

An NRPS gene cluster in scaffold 03 encodes orthologues (ORF 606 to ORF 611) of *mxcC*, *mxcD*, *mxcE*, *mxcF*, and *mxcG*, myxochelin-biosynthetic genes in *Stigmatella aurantiaca* Sg a15 (2). This indicates that the NRPS gene cluster is responsible for myxochelin A biosynthesis in *Nonomuraea* sp. TP-A0861. Except for the NRPS gene cluster, the genome contains two orphan NRPS gene clusters in scaffold 01. One (ORF 821 to ORF 829) contains eleven NRPS modules and the other (ORF 888 to ORF 891) two modules (7), suggesting that their products are an oligopeptide composed of 11 amino acids and a dipeptide, respectively. Additionally, two type I PKS gene clusters (scaffolds 02 and 07), one type II PKS gene cluster (scaffold 07), and one hybrid PKS/NRPS gene cluster (scaffold 01) are present in the genome. Sequence similarities of these gene clusters are low to the gene clusters of known secondary metabolites.

Recently, draft genome sequences of *Nonomuraea candida* NRRL B-24552 (JOAG00000000.1), *Nonomuraea coxensis* DSM 45129 (ARBV00000000.1), and *Nonomuraea kuesteri* NRRL B-24325 (JOAM00000000.1) were released to the public. However, no scientific papers regarding these strains have been published yet. Therefore, this is the first report of the genome sequence of the genus *Nonomuraea*. The genome sequence of *Nonomuraea* sp. TP-A0861 will provide valuable information to elucidate the potential of *Nonomuraea* strains as a source of new bioactive compounds.

### Nucleotide sequence accession numbers.

The draft genome sequence of *Nonomuraea* sp. TP-A0861 has been deposited in the DDBJ/ENA/GenBank database under the accession number BBZG00000000. The version described in this paper is the first version, BBZG01000000.
